# Response: Commentary: Severe Sequelae to Mold-Related Illness as Demonstrated in Two Finnish Cohorts

**DOI:** 10.3389/fimmu.2018.01220

**Published:** 2018-06-11

**Authors:** Tamara Tuuminen, Kyösti Rinne

**Affiliations:** ^1^Medicum, Department of Bacteriology and Immunology, University of Helsinki, Helsinki, Finland; ^2^KristinaMedi Oy, Kauhajoki, Finland

**Keywords:** sick building syndrome, autoimmune conditions, multiple chemical syndrome, malignancies, environmental molds, indoor air, hypothyroidism, mold-related illness

We thank Heinzow HS and Heinzow BGJ for their interest toward our paper.

Indeed, we are glad that our paper caused useful academic debates.

First, it is important to emphasize that ours was a case report and a retrospective study. We did not claim that we were presenting an epidemiological study with an enrollment of well defined and matched study and control groups with inclusion and exclusion criteria, etc. In fact, we really do not know how such a study aiming to investigate the association of moldy building with cancer could be designed in an ethical way. These studies are important but methodologically very challenging, long lasting, and require resource, awareness, and an open mind. Because we presented a case report, statistical treatment of the data were not applicable. As it usually happens in medicine, somebody presents the description of the phenomenon or the clinical picture of an unknown disease usually as a case report. Later, this report creates more interest with an outcome of new studies, including epidemiological ones. Without investigation into the phenomena, epidemiological studies will not be initiated. Our report was to highlight that this association should not be overlooked and should be studied with all appropriate methods.

Second, we did mention our study limitation. We emphasized that our observation is based on higher than average morbidity and mortality among students and teachers who were compared to the morbidity in the area using available registers.

Next, we want to make short comments on both cohorts.

The first cohort. All the family members living in a moldy building in addition to symptoms already described in the literature and cited by the authors of the commentary suffered from Multiple Chemical Sensitivity. As far as we know, such an association has not yet been reported, however, in our clinical practice, patients complain about acquired hypersensitivity to many chemical substances that they could tolerate before. We are convinced that such an association should be reported.

In Finland, the Dampness and Mold Hypersensitivity Syndrome (DMHS) is very prevalent. The official estimation is that approximately 12–18% of the schools and kindergartens are water damaged and that approximately 172,000–259,000 of persons are exposed to dampness microbiota (1). However, DMHS is currently considered a functional impairment that gives no social security.

The authors of the commentary accurately note that DMHS is acknowledged in Finland, which has a comparatively large research output on this topic as compared to other countries. Despite this research, it does not appear to translate into public heath actions, and we feel that this must be addressed.

The second cohort. This cohort describes a cluster of rare autoimmune diseases and oncologic morbidities with increased mortality. Depending on the readers experience and attitudes, some may interpret this morbidity surge as occurred only by chance. Other readers may not believe in chance but may suspect the causality between air toxicity and morbidity. Mycotoxins have very many actions on the cell structures, and their functions have been reviewed in very many publications, e.g., Ref. (2).

The action of mycotoxins on the cellular NRLP3 inflammasome was compared to tobacco and ricin, the latter being a chemical warfare (3). From that perspective it seems illogic not to consider mycotoxins as hazardous compounds. The authors of the commentaries are wrong stating that “*mycotoxins are not volatile but bound to spores and hyphen fragments*.” Mycotoxins are volatile. Recently, the terminology of “volatoxins” (volatile toxins) has been suggested (4).

The measurement of air toxicity better than the isolation techniques correlates with morbidities. Conventional techniques have limitations: isolation of microbes is a tedious procedure especially of the slowly growing species; some isolates may be more toxic than others that will not be reflected in isolation results only. Calculation of microbial particles neither may reveal health hazards. A cross-sectional study showed that air toxicity correlates well with the morbidity in school teachers (5). Mycotoxins do have a tendency to be aerosolized [(6), reports from Indoor Climate Seminars, Helsinki, Finland: (7, 8)].

In the school, the following dampness microbiota species were isolated, many of them are potential mycotoxin producers:

Konto^®^ insulation: *Actinomycetes, Asperrgillus fumigates, Aspergillus flavus, Aspergillus peniccilloides/restrictus, Tritirachium, Penicillium, Exophiala*.
Cellulose wool: *Actinomycetes, Aspergillus fumigatus, Penicillium, Paecilomyces* species.
Glass wool: *Aspergillus* species, *Streptomyces* species, etc.

The investigation of the toxicity studies was performed on June 16, 2017 by addition of the condensed water to the culture of human macrophages (9). The results were the following: classroom K1: 5.3 ± 3.8% of dead macrophages, *p* = 0.007; small classroom K2: 6.90 ± 3.30%, *p* < 0.001. The toxicity was significant compared to the sterile water control. These results were obtained in the accredited laboratory FICAM; worldwide patent for the sample collection method was applied.

Furthermore, the answers to the comments are the following:

1. ICD-10 codes for all the patients are available, but usually in scientific literature and in a report like ours, these are not mentioned. Besides, due to the small size of community where the school is located, we could not open the codes to make impossible identification of the patients.
2. Iodine deficiency to explain hypothyroidism is too farfetched. Iodine is supplemented to consumer salt. Again, here we compared the cluster prevalence to the average. This difference cannot be explained by micronutrient deficiency.
3. We checked the meteorological reports after the Tschernobyl catastrophe. The radioactive cloud was in this area but according to statistics this did not increase mortality and morbidity from cancer in the area.

All in all, we agree that the association of mycotoxins from indoor molds and cancer should be proven in carefully designed but ethical studies. Our report was an impetus to go this way.

## Author Contributions

All authors listed have made a substantial, direct, and intellectual contribution to the work and approved it for publication.

## Conflict of Interest Statement

The authors declare that the research was conducted in the absence of any commercial or financial relationships that could be construed as a potential conflict of interest.
